# Weighted Blanket Therapy for Periodic Limb Movement Disorder: A Case Report Highlighting Improved Sleep Quality and Reduced Symptoms

**DOI:** 10.7759/cureus.39622

**Published:** 2023-05-29

**Authors:** Muhammad Nouman Aslam, Riju Kafle, Saima H Shawl, Armughan S Khan, Muhammad Waseem Kagzi

**Affiliations:** 1 Internal Medicine/Sleep Medicine, Midwest Sleep & Wellness Clinic, Chicago, USA; 2 Sleep Medicine, Midwest Sleep & Wellness Clinic, Chicago, USA; 3 Sleep Medicine, Advocate Condell Medical Center, Chicago, USA

**Keywords:** sleep disorders, non-pharmocologic intervention, sleep medicine, sleep quality, weighted blankets, sleep hygeine, periodic limb movements

## Abstract

This case report describes a 20-year-old female patient with periodic limb movement disorder (PLMD), who experienced trouble sleeping and daytime fatigue. Polysomnography revealed frequent non-arousing periodic limb movements and a high PLMD index. The patient was advised on non-pharmacological interventions, including the use of weighted blankets, sleep hygiene education, and lifestyle modifications. At the six-week follow-up, the patient reported significant improvement in symptoms. The case report highlights the potential effectiveness of non-pharmacological interventions in managing PLMD and emphasizes the need for a multidisciplinary approach to improve patient outcomes and quality of life. Further research is required to determine the long-term efficacy and safety of these interventions. The psychological impact of PLMD on the patient's social life and academic performance is also discussed. The management of sleep disorders should involve a multidisciplinary approach to improve patient outcomes and quality of life.

## Introduction

Periodic leg movement syndrome (PLMS), a sleep disorder that can disturb sleep and lead to excessive daytime sleepiness (EDS), is characterized by recurrent, unconscious leg movements such as repetitive jerking, cramping, or twitching of the lower limbs during sleep [1]. Up to 4% of the general population may be affected by PLMS, and it is particularly prevalent in the elderly [2].

PLMS has been associated with a variety of underlying conditions, including restless leg syndrome (RLS), attention-deficit/hyperactivity disorder (ADHD), and multiple sclerosis [3]. The exact cause of PLMS is not well understood, but research suggests that in the case of primary periodic limb movement disorder (PLMD), abnormalities in the central nervous system and certain neurotransmitters may play a role, whereas, secondary PLMD may be attributed to diabetes, iron deficiency, caffeine use, spinal cord injury or tumor, uremia, and anemia [4]. Also, certain genes like MEIS1 and BTBD9 linked with RLS may be associated with the incidence of PLMS [5].

Effective treatment options for PLMS include certain medications - alpha 2-delta ligands such as gabapentin; dopaminergic agents like pramipexole and ropinirole - as well as lifestyle changes, and behavioral therapy such as reducing caffeine and alcohol intake, better sleep hygiene, deep breathing exercises, meditation or yoga, and incorporating more iron into the diet. It is important for individuals experiencing symptoms of PLMS to seek medical attention and obtain a proper diagnosis to determine the underlying cause and initiate the appropriate treatment [2]. We present a case of a 20-year-old female patient whose PLMS was managed with weighted blankets. As there is limited literature discussing the use of weighted blankets in managing PLMS, we believe our case report will provide an impetus for clinicians and researchers to investigate the mechanisms by which they might play a role in the treatment.

## Case presentation

A 20-year-old female with a medical history of PLMD and an enlarged thyroid was referred to our sleep clinic due to complaints of trouble sleeping and daytime fatigue. The patient reported difficulty falling asleep, frequent awakenings, trouble going back to sleep, and repetitive leg movements throughout the night. These symptoms resulted in fragmented and poor-quality sleep, causing daytime fatigue that sometimes interfered with her college routine. Additionally, her jerking limb movements had led her roommate to complain, prompting her to move to a single room. The patient's college life was affected by her PLMD, resulting in a change in living arrangements that caused additional stress and anxiety.

On the first visit, a physical examination revealed an enlarged thyroid. Polysomnography revealed frequent non-arousing PLMS with a PLMS index (periodic limb movements per hour) of 665 per hour and a PLMS arousal index of 11.9 per hour (Table 1). Further investigation results were within normal limits, including thyroid-stimulating hormone (TSH), iron, total iron-binding capacity (TIBC), and ferritin.

**Table 1:** Leg movement summary from the polysomnography results of the patient

	Number	Number per hour
Leg movements	738	127.8
Periodic leg movements	665	665
Periodic leg movement arousals	69	11.9

A treatment plan was devised, which included advising the patient to sleep in a cool area and exercise in the early morning. The patient was also advised to cut down on sugar and caffeine intake, educated on sleep hygiene, and offered medication options, such as pramipexole, gabapentin-enacarbil, melatonin, and magnesium. However, the patient had tried melatonin before with no success. The patient was also advised to use weighted blankets to alleviate symptoms.

At the six-week follow-up, the patient showed significant improvement. Compliance with weighted blankets and sleeping in a cooler room led to better sleep quality, and no medication changes were initiated. Improvement in sleep quality also likely accounted for a positive impact on the patient's mood.

## Discussion

PLMD is a sleep disorder characterized by repetitive limb movements during sleep that can lead to fragmented sleep and daytime fatigue. PLMD is often associated with RLS, a neurological disorder characterized by an irresistible urge to move the legs. PLMD can occur in both children and adults and is often associated with other medical conditions, such as thyroid disorders, iron deficiency anemia, and renal failure [6].

In our case, the patient had a medical history of PLMD from childhood and an enlarged thyroid. She reported trouble sleeping, daytime fatigue, and repetitive leg movements throughout the night, which significantly impacted her daily life and college routine, leading to a change in living arrangements. PLMD not only has physical effects but also has a significant psychological impact on patients. Poor sleep quality and daytime fatigue can lead to mood disturbances and conditions such as depression and anxiety [7]. This case highlights the importance of addressing both the physical and psychological aspects of PLMD, including its social impact on patients [8].

The diagnosis of PLMD is made through a combination of clinical history and polysomnography. Polysomnography is the gold standard for diagnosing PLMD, as it allows for the detection of periodic limb movements and the assessment of their impact on sleep quality [9]. In our case, polysomnography revealed frequent non-arousing PLMS, with a PLMS index of 115.2 per hour (diagnosis requires PLMS index >15) and a PLMS arousal index of 11.9 per hour. The PLMS index is used to gauge PLMS severity. A PLMS index of more than 50 is considered to indicate severe PLMD [10].

The patient had normal sleep architecture and sleep efficiency (Table 2). In addition, there were no significant apnea and hypopnea events during the patient's polysomnography study (Table 3). Utilizing clinical signs and sleep study data, a diagnosis of PLMD was made.

**Table 2:** Sleep architecture during the sleep study REM: rapid eye movement

Sleep architecture portion of the study	
Total sleep time	346.5 min
Total time in bed	410 min
WASO (wake after sleep onset)	40.8 min
Sleep efficiency	84.5%
Latency to sleep onset	22.7 min
Latency to REM onset	234.2 min

**Table 3:** Respiratory events during the sleep study RERAs: respiratory effort-related arousals

Respiratory events	Number per hour
Apneas	0
Hypopneas	1
Total apneas/hypopneas	1
Apnea/hypopnea index (AHI)	0.2
RERAs index	4.0
Respiratory disturbance index (RDI = AHI + RERAs)	4.2

Treatment options for PLMD include both non-pharmacological and pharmacological interventions. Non-pharmacological interventions include sleep hygiene education, regular exercise, and the use of weighted blankets. Weighted blankets have been shown to reduce the frequency of leg movements and improve sleep quality in patients with PLMD [11]. Weighted blankets work on the principles of deep pressure therapy (DPT) [12], which has an inhibitory effect on the sympathetic nervous system, thereby producing a calming effect for the patient [13]. One study found that using weighted blankets increased the release of melatonin and oxytocin, both of which help with stress relief [14]. As patients with PLMD also have associated sleep disturbance, the relaxing effect of this intervention helps patients overcome the distressing effects of the disease on their sleep.

Pharmacological interventions for PLMD include dopaminergic agents such as pramipexole and ropinirole, which have been shown to reduce the frequency of leg movements and improve sleep quality. However, these medications may have side effects such as nausea, dizziness, and headache. The use of benzodiazepines and opioids should be avoided due to the risk of addiction and other side effects [15].

In 2020, a study in the Journal of Clinical Sleep Medicine found that using actigraphy to monitor and improve sleep hygiene behaviors led to significant improvements in sleep quality and reduced limb movements in PLMD patients [16]. In 2021, a Sleep Medicine study found that acupuncture effectively reduced limb movement frequency and severity, apart from improving sleep quality [17]. Another 2021 Sleep Medicine Review study found that cognitive behavioral therapy (CBT) was effective in improving sleep quality and reducing PLMD symptoms in patients with comorbid insomnia and PLMD [18]. These studies suggest that non-pharmacological interventions, such as acupuncture, CBT, and the use of wearable technology, can be effective in improving sleep quality and reducing symptoms in patients with PLMD.

In our case, the patient was advised to sleep in a cool area, exercise in the early morning, and reduce her sugar and caffeine intake. She was educated on sleep hygiene and offered medication options, such as pramipexole, gabapentin-enacarbil, melatonin, and magnesium. She had tried melatonin previously but experienced no improvement in her sleep quality. Therefore, the patient expressed her preference for a non-pharmacological approach. Weighted blankets were suggested as an option and upon adhering to this non-pharmacological intervention over the next six weeks, the patient reported a substantial improvement in her sleep quality without requiring any medication adjustments.

## Conclusions

PLMD is a sleep disorder that can have a significant impact on the patient's quality of life, including the psychological and social aspects. A multidisciplinary approach to the management of PLMD, addressing both the physical and psychological aspects of the disease, is crucial to achieving the best possible outcomes for the patient. Non-pharmacological interventions, including sleep hygiene education and the use of weighted blankets, might be considered first-line treatment options. The patient in this case had experienced a significant impact on her daily college routine and had to change her living arrangements due to her condition. Therefore, healthcare providers should consider the psychological impact of PLMD and provide appropriate support and resources to patients. Non-pharmacological interventions such as weighted blankets have not been studied extensively. In our case of severe PLMD, weighted blankets alleviated patient symptoms effectively, and this method can be employed in patients hesitant toward medical therapy. Overall, a comprehensive and individualized approach to managing PLMD is necessary to ensure optimal patient outcomes, with a focus on both the physical and psychological aspects of the disease.
